# Imaging Findings of Bisphosphonate-Related Osteonecrosis of the Jaws: A Critical Review of the Quantitative Studies

**DOI:** 10.1155/2014/784348

**Published:** 2014-06-11

**Authors:** André Ferreira Leite, Fernanda dos Santos Ogata, Nilce Santos de Melo, Paulo Tadeu de Souza Figueiredo

**Affiliations:** ^1^Oral Radiology, Department of Dentistry, Faculty of Health Science, University of Brasília, Campus Universitario Darcy Ribeiro, Asa Norte, 70910-900 Brasília, DF, Brazil; ^2^University of Brasília, Campus Universitario Darcy Ribeiro, Asa Norte, 70910-900 Brasília, DF, Brazil; ^3^Oral Pathology, Department of Dentistry, Faculty of Health Science, University of Brasília, Campus Universitario Darcy Ribeiro, Asa Norte, 70910-900 Brasília, DF, Brazil

## Abstract

*Objectives*. This paper offers a critical review of published information on the imaging strategies used for diagnosing bisphosphonate-associated osteonecrosis of the jaw (BRONJ) in patients taking intravenous bisphosphonates, pointing at the different methodologies and results of existing literature. *Methods*. Electronic literature search was performed in order to identify as many quantitative studies that discussed the imaging findings of BRONJ up to February 2014. Initially, the search for articles was based on the following four types of imaging modalities for evaluating BRONJ: computed tomography, plain film radiographs, magnetic resonance imaging, and nuclear bone scanning. *Results*. Eleven out of the 79 initially selected articles met the inclusion criteria. Most of the selected articles were cross-sectional studies. Regarding the selected studies, 54.5% have used plain films radiographs and 54.5% were based on computed tomography findings. All of the selected studies showed a small number of patients and none of the selected studies have tested the accuracy of the imaging examination for evaluating BRONJ. *Conclusions*. This critical review showed a scarcity of quantitative studies that analyzed the typical imaging findings related to BRONJ. Further studies are necessary in order to analyze the role of different imaging techniques in the assessment of BRONJ.

## 1. Introduction

Bisphosphonates are the first line of treatment for metastatic bone cancer, osteoporosis, and Paget's disease. In the late 2003, cases of bisphosphonate-related osteonecrosis of the jaw (BRONJ) were first reported [1]. Since then, many studies have been performed in order to provide early diagnosis and better treatment for the patient once the BRONJ negatively affects their quality of life and increases morbidity. The cumulative incidence of BRONJ in patients taking intravenous bisphosphonates is significantly greater than in patients using oral bisphosphonates and varies from 0.8% to 12%. The estimated risk of BRONJ for oral bisphosphonate users remains uncertain but the occurrence appears to range from 1 in 10 000 to 1 in 100 000 patient-years [2–4].

The American Academy of Oral and Maxillofacial Surgeons stated that, for the clinical diagnosis of BRONJ, patients need to exhibit all of the following three characteristics: (1) current or previous treatment with a bisphosphonate; (2) exposed, necrotic bone in the maxillofacial region that has persisted for more than eight weeks; and (3) no history of radiation therapy to the jaws [2].

BRONJ is categorized according to the clinical signs and symptoms into stage I, stage II, and stage III. Clinically, the disease appears as a nonhealing exposed bone area that can be accompanied by fistulization, purulent discharge, and pain [5]. Although imaging findings neither are considered diagnostic criteria nor have radiographic features for each stage, their findings corroborate the evaluation of the course, extent, and progression of the disease. The clinical examination does not usually show the full extent and severity of BRONJ sites beneath the mucosa [6]. Panoramic radiography, computed tomography (CT), magnetic resonance imaging (MRI), and scintigraphy are valuable imaging modalities that assist the clinical findings by revealing different aspects of bone involvement. Furthermore, these imaging examinations can help in the differential diagnosis of other diseases that resemble BRONJ in terms of clinical signs and symptoms [7–9].

Radiographic exam is additionally substantial since most patients with BRONJ are those undergoing other treatments and the imaging findings of BRONJ are not specific and can also be found in other conditions such as osteomyelitis, osteoradionecrosis, cancer metastasis, and Paget's disease [10]. The initial imaging findings in BRONJ appear to be focal medullary sclerosis with poor corticomedullary differentiation, which is clinically concomitant with the loosening of tooth. A usual sign of osteonecrosis of the jaw is the delayed socket healing after tooth extraction. In late disease, there is a sequestrum formation, fractures, and reaction, and when the maxilla is involved, there may be mucosal thickening in the adjacent sinus with fluid levels or purulent discharge [4].

Despite the lack of consensus on the radiographic evolution of BRONJ, the literature has shown through models the formation of a necrotic body or involucrum inside the trabeculae in sclerotic mandibular bone. The involucrum represents most likely dead bone, which becomes surrounded by a resorptive circumference that increases with time. Probably, this is a response by the bone cells to remove the dead bone. The involucrum follows the path of least resistance leading to an exposed sequestrum or, if the tooth is missing moves to the edentulous area, suggesting that this could be the mechanism of the formation for the clinically visible sequestrum [11].

A major challenge is the early diagnosis of BRONJ lesions, preferably when still there is no exposed bone, which allows better treatment and prevention of exposures. Therefore, studies that aimed to diagnose by imaging examinations the bone changes that precede the clinical alterations are shown to be of great value. In this regard, some authors have demonstrated the presence of regional bony sclerosis similar to cases of stages 1 to 3 BRONJ in patients characterized as stage 0 BRONJ [12].

Several imaging features of BRONJ have been previously reported [5–22], including bone sclerosis, widening of the periodontal ligament space, cortical surface irregularities, persistent extraction sockets, bone fragmentation (sequestration), and osteolytic changes. However, the frequency and consistency of these findings and the correlation between imaging and clinical findings remain unclear. The correlation between imaging findings and the temporal development of BRONJ is also unclear. Therefore, this paper offers a critical review and analysis of published information on the imaging quantitative studies for BRONJ patients, pointing at the different methodologies and results of existing literature.

## 2. Methodology

### 2.1. Search Strategy

Electronic literature search was performed in order to identify as many quantitative studies as possible that analyzed the imaging findings of BRONJ up to February 2014. Databases including Pubmed/Medline, Scielo, Cochrane's Reviews, and Scopus were searched in English.

**Figure 1:**
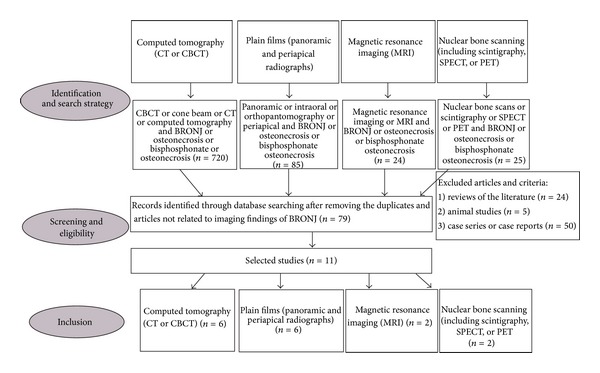
Flow chart of the study selection procedure.

Initially, the search for articles was based on the type of imaging examination. For this purpose, the imaging modalities for evaluating BRONJ were divided into the following four groups: (1) computed tomography (CT), including both multidetector computed tomography (MDCT) and cone beam computed tomography (CBCT); (2) plain films, including panoramic and intraoral radiographs; (3) magnetic resonance imaging (MRI); (4) nuclear bone scanning, including scintigraphy, SPECT, or PET.  Figure 1 shows the flow chart of the study selection procedure.

Reports of any study design (clinical trials, cohort, case-control, and cross-sectional studies) were included investigating the imaging strategies used for diagnosing bisphosphonate-associated osteonecrosis of the jaw in patients taking intravenous bisphosphonates. All studies that performed quantitative analyses were included. The final selection was completed after eliminating the duplicated articles, case reports, case series, reviews of the literature, editorials, anecdotal letters, letters to the editors, and those articles that were not related to imaging findings for evaluating BRONJ patients.

### 2.2. Statistical Analysis

Statistical analysis of data from the selected studies was not attempted due to the variations in the study design, methodology, and choice of imaging modality.

## 3. Results

From the initial search, most of the excluded articles were not related to imaging findings of BRONJ. After eliminating the duplicated articles and those that were not related to imaging findings of BRONJ, the initial database search yielded 79 different abstracts. Nevertheless, only eleven of these initially selected studies met the inclusion criteria [6, 11, 13–21].

**Figure 2:**
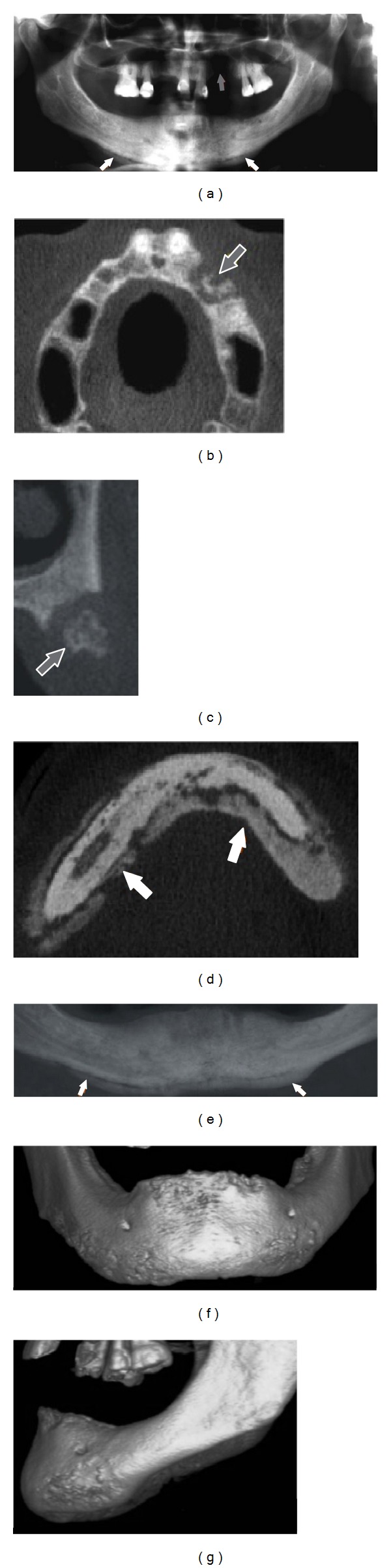
Imaging findings of a 57-year-old woman with metastatic breast carcinoma receiving intravenous zoledronic acid. (a) Panoramic radiograph showing maxillary involvement with radiographic evidence of osteolysis (gray arrow). (b) and (c) axial and cross-sectional CBCT views, respectively, showing the necrotic area with bone sequestrum in the left maxilla (gray arrow). (d) Axial CBCT image showing the extent of mandible bone involvement with periosteal bone reaction. The periosteal bone reaction changed the mandibular morphology, as it can be seen in the two-dimensional multiplanar reconstruction image ((e), white arrow) and in the 3D images (frontal view (f) and sagittal view (g)).

**Figure 3:**
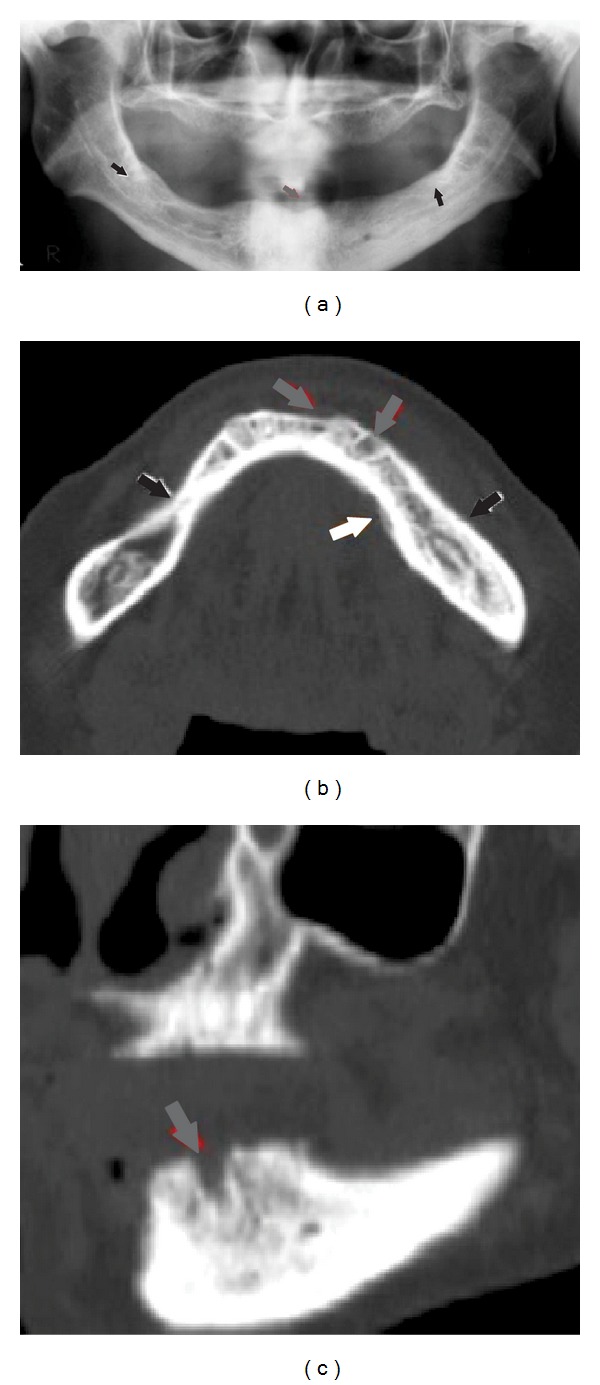
Imaging findings of a 65-year-old woman with metastatic breast carcinoma receiving intravenous zoledronic acid. (a) Panoramic radiograph showing an osteolytic lesion in the anterior mandible (gray arrow) and areas of osteosclerosis in the posterior regions (black arrows). (b) Axial CBCT image reveals areas of osteolysis (gray arrows), areas of osteosclerosis (black arrows), and a periosteal bone reaction in the left mandible (white arrow). (c) Sagittal CBCT image demonstrates a nonhealing extraction socket in the anterior mandible.

Regarding the excluded articles from the second search, most of the studies were case series/reports of cases (63.3%) that only described imaging features of BRONJ patients. Figures 2 and 3 show examples of the main imaging findings of BRONJ in two patients taking intravenous zoledronic acid. Furthermore, five excluded studies were performed in animals (6.3%) and 30.4% were reviews of the literature.

### 3.1. Characteristics of Included Papers

Information on the study patient's demographics, study design, imaging modalities, and technical parameters of the eleven included papers is outlined in Table 1.  Table 2 shows the objectives, main results, and main conclusions of each selected studies.

Regarding the selected studies, 54.5% (6 studies) have used plain films radiographs and 54.5% (6 studies) were based on computed tomography findings. Only two quantitative studies were found with MRI (18.2%) and with nuclear bone scanning (18.2%).

## 4. Discussion

As far as we know, the present study is the first critical review aiming at discussing little evidence about imaging findings of BRONJ. Initially, we intended to perform a meta-analysis of the existing literature regarding imaging modalities for BRONJ patients. However, due to the scarcity of quantitative studies with a similar methodology, it was only possible to perform a critical review and qualitative analysis of the published studies related to this issue.

In our review, many studies (63.3%) were retrospective case series or case reports with unclear incidences and frequency estimates of imaging findings. For this reason, this kind of studies entered in the exclusion criteria of our review. An attempt has been made to collate, compare, and discuss the methodology and results of different studies that quantitatively evaluated the imaging findings of BRONJ in patients taking intravenous bisphosphonates. The reading of these selected studies showed a significant heterogeneity. In addition to the small amount of selected articles, the comparison of the findings was difficult due to the significant methodological differences between each study, conflicting results, small sample sizes, and the variability of imaging techniques. Furthermore, the absence of diagnostic test studies that report the specificity and sensitivity precluded the analysis of accuracy of each imaging modality.

Few studies have evaluated imaging findings in bisphosphonate-treated patients with stage 0 disease in the absence of bone exposure [11, 12]. The former was a prospective study conducted with clinical and dental panoramic analysis of 60 patients. Of these 60 patients, thirty were treated with zolendronate and 30 composed the control group. Patients treated with the intravenous aminobisphosphonate presented a statistically significant increase in the number of radiographic abnormalities compared with the control group. However, this selected study has not described or discussed the radiographic findings. The second aforementioned study analyzed patients receiving oral bisphosphonate therapy which is not the main risk group for developing BRONJ. As this study was only descriptive, it was excluded from our sample.

Diagnosis of BRONJ is usually made at the late stage when there is bone exposure to the oral cavity. Standard diagnosis based on clinicoradiological criteria is still lacking and there are no clinicoradiological guidelines for the health professionals to follow. In our systematic review, four of the eleven selected studies have used exclusively plain films such as panoramic and periapical radiographs [11, 14, 17, 20]. However, these studies have different objectives and methodologies and different patient populations and types of bisphosphonate therapies, which preclude a direct comparison of their results. Some authors have stated that a higher risk of developing BRONJ apparently may be predicted detecting the rise of alveolar bone mineral density that frequently occurs near the necrotic lesion [17] and by the presence of a radiographic periodontal ligament widening [14].

Dental panoramic radiograph and computed tomography can be considered as the most widely available imaging techniques for BRONJ evaluation. This can explain why most of the selected studies have used those imaging modalities [11, 13–16, 18–20]. Furthermore, they usually detect dentoosseous changes related to this entity, including bone sclerosis, cortical surface irregularities, persistent extraction sockets, bone fragmentation (sequestration), and osteolysis.

Despite being the most used imaging modalities for BRONJ evaluation, there are some contradictory results on the selected studies. Some authors have suggested that panoramic radiographs are useful for evaluating BRONJ [11, 20]. On the other hand, other authors have stated that these radiographs are of limited value for this purpose [13, 16]. The differences may be related to the imaging modalities used in the studies. The selected studies that emphasized the role of the plain film radiographs for BRONJ evaluation have not used 3D images [11, 14, 17, 20]. On the other hand, the criticism of some authors regarding plain film radiographs was based on comparison with other 3D imaging modalities such as CT and MRI [16, 20]. Panoramic radiograph may be a useful and readily accessible imaging examination for the initial radiologic investigation in patients treated with intravenous bisphosphonates. This kind of radiography allows quick visualization of the entire affected area and seems to be able to demonstrate clear signs of osteolytic lesions mainly when radiopaque sequestra are present or when osteolysis is combined with osteosclerosis [5, 7]. In a previous cross section study with 39 patients, a correlation was found between focal panoramic radiographic findings of bone sclerosis and surface irregularity with clinical sites of BRONJ [20]. However, the disadvantages of panoramic radiograph should be recognized, such as missing definition among the margins of the necrotic areas and healthy bone, the difficulty in distinguishing osteonecrosis of a malignant lesion when an osteolytic lesion is present, and the limited image in a two-dimensional view of three-dimensional structures. Such limitations restrict the understanding of all the extent of the lesion [5, 14]. As a conventional radiograph, panoramic images often suffer from magnification, distortion, and superimposition. Moreover, a successful panoramic radiograph requires careful positioning of the patient and proper technique. Therefore, the limitations of this imaging modality for BRONJ patients should be emphasized, especially in elderly or noncollaborating patients [23, 24].

Computed tomography (including multidetector CT or CBCT) has been demonstrated to be superior to panoramic in detection and evaluation of BRONJ, particularly with regard to soft tissue swelling, new bone, and sequestrum [13, 16]. CBCT may also be used for detection of bone alterations by evaluating the fractal dimension of the alveolar process [18] and measuring the mandibular cortical bone that are higher in BRONJ patients [19]. CBCT may also allow the detection of subclinical, small involucra and has potential in monitoring the progression of the lesions [25]. Compared with multidetector CT, CBCT is easy to use, with short acquisition scan times and high resolution, can be performed while patients are in the upright position, and is of low cost [26].

Our systematic review has shown that the selected studies have used different imaging modalities such as periapical radiographs [14, 17], panoramic radiographs [11, 13, 14, 16, 20], multidetector computed tomography [13, 16], cone beam computed tomography [15, 18, 20], MRI [15, 16], PET/CT [15], and scintigraphy and SPECT [21]. Apparently, CT scan is extremely useful in defining the features and extent of the lesions and, in selected cases, an MRI can add value to the radiological findings by showing the soft tissue involvement. However, there have been no studies that have rigorously compared these various modalities for their utility in evaluating BRONJ, especially regarding clinically relevant end points [22].

Although imaging examination can be very useful in determining the extent of bony changes, only one selected study has compared different imaging modalities for this purpose [15]. PET/CT and MRI revealed more extensive involvement of BRONJ compared with CBCT and clinical examinations. However, only 10 patients have been evaluated in this prospective cross-sectional study. Further prospective studies are necessary to verify which imaging modality is better for evaluating the extent of BRONJ. The role of nuclear bone scanning for evaluating patients taking intravenous bisphosphonates also deserves further investigation. In a cohort study with 22 subjects, some authors have demonstrated that the relative quantification of tracer uptake provides prognostic information independent of clinical stage of BRONJ [21]. Although scintigraphy is a very sensitive investigation it may be used as a screening test to detect subclinical osteonecrosis in patients receiving bisphosphonates [7, 27], but it should be kept in mind that the rate of false positives may be high due to the lack of specificity [28].

This study has its own limitations. Due to the scarcity of the literature it was not possible to compare quantitatively the selected studies. Consequently, it was decided to select all the quantitative studies, despite of the significant differences in methodologies, imaging modalities, kind of studies, and populations. Although several theories about the etiology of the BRONJ have been advanced, many questions remain unanswered, especially regarding the pathophysiology [3]. The complete understanding of the pathogenesis may also contribute to the development of prevention and treatment guidelines, including the guidelines for prescription of imaging examinations.

In conclusion, this critical review showed a scarcity of quantitative studies that analyzed the typical imaging findings related to BRONJ. Further studies are necessary in order to analyze the frequency and how the typical findings appear, and also the timing of their appearance. Clinical guidelines for BRONJ need to include which imaging modality should be performed for BRONJ patients and at what time intervals. Although conventional radiographs can demonstrate evidence of BRONJ, especially when disease is advanced, there are limitations of these imaging modalities, regarding their 2D nature and also the technical characteristics. While CBCT scans provide more information regarding the extent of bone changes, the usefulness of this imaging modality in asymptomatic individuals should be better investigated. Further study would be useful to identify, based on clinical and radiographic factors, whether CBCT examinations are justified for all BRONJ patients. Nuclear medicine modalities, such as PET/CT, may also be considered as promising tools for BRONJ evaluation. Diagnostic test studies and the comparison of the various imaging modalities are still necessary.

## Figures and Tables

**Table 1:** Study design, population characteristics, imaging methods, equipment, and set conditions of each selected study.

Authors (year)	Study design	Population characteristics	Imaging method	Equipment, contrast medium or radionucleotide, and set conditions
Bianchi et al., 2007 [13]	Cross-sectional	32 subjects (20 women; range: 48–84 years)	MDCT + PAN	PAN: Orthophos (Sirona, Bensheim, Germany; at 69–71 kV and 15 mA for 14.2 s)/MDCT: Lightspeed Pro 16 and Lightspeed QX/i (GE, Milwaukee, WI; at 120 kV and 80–120 mA, 0.6 mm slice thickness, 0.9 pitch, and 12.8 cm FOV)

Fleisher et al., 2010 [14]	Case-control	68 subjects (gender and year's range: N/E)	PER + PAN	N/S

Guggenberger et al., 2013 [15]	Cross-sectional	10 subjects (9 women; mean age 69,6; range: 53–88 years)	MRI + PET/CT + CBCT	MRI: 1.5 T scanner (Signa Excite HDxt; GE Healthcare; Milwaukee, WI). An 8-channel transmit-receive head coil was used. PET/CT: (Discovery RX or Discovery STE; GE Healthcare). CBCT: KaVo 3D eXam (KaVo, Biberach, Germany) with an amorphous silicium flat panel detector (20 × 25 cm). Exposure volume: 102 mm. Voxel size: 0.4 mm. The scan was set at a high-frequency constant potential of 120 kV (peak)

Rocha et al., 2012 [11]	Cohort	60 subjects; 30 cases and 30 controls (case: 18 women; range: 41–91 years/control: 22 women; range: 50–64 years)	PAN	Planmeca machine (Proline XC Digital model, 78 kV and 10 mA/18 s)

Stockmann et al., 2010 [16]	Cross-sectional	28 subjects (16 women; range: 57–78 years)	PAN + MRI + MDCT	PAN: Orthophos TM, Sirona, Bensheim, Germany, ×1.2 magnification; with gender specific settings (female patients, 69 kV and 15 mA; male patients, 66 kV and 8 mA)/MRI: 1.5 T (Magnetom Symphony TM, Siemens, Erlangen, Germany)/MDCT: 64-slice MDCT-Scanner (Somatom Sensation 64TM, Siemens, Forchheim, Germany). Scan settings were 120 kV, 110 mAs eff., 64 × 0.6 slice acquisition, 0.9 pitch, 1 s rotation time, 1 mm reconstructed slice thickness, 0.8 mm reconstruction increment, and sharp kernel (B70s)

Takaishi et al., 2010 [17]	Cross-sectional	48 subjects; 6 cases and 42 controls; age-matched (case: gender N/E; range: 47–75; control: gender N/E; range: 45–76 years)	PER	N/S

Torres et al., 2011 [18]	Cross-sectional	36 subjects; 9 cases and 27 controls; gender- and age-matched (case: gender N/E; range: 43–83 years; control: gender N/E; 43–84 years)	CBCT	MercuRay**©** CBCT System (Hitachi Medical Corporation, Tokyo, Japan)

Torres et al., 2012 [19]	Cross-sectional	58 subjects; 10 cases and 48 controls; gender- and age-matched (case: 7 women; range: 49–77 years; control: 34 women; range: 49–76 years)	CBCT	CB MercuRay equipment (Hitachi Medical Corporation, Tokyo, Japan)

Treister et al., 2009 [20]	Cross-sectional	39 subjects (15 women; range: 40–83 years)	PAN	N/S

Van den Wyngaert et al., 2011 [21]	Cohort	22 subjects (19 women; range 48–78 years)	SCI + SPECT	SCI: intravenous administration of medronate (methylene diphosphonate (MDP)) labeled with 740 MBq (20 mCi) Tc-99 m (Amerscan Medronate II Agent, GE Healthcare Limited, UK)/SPECT: step-and-shoot mode was used to obtain 64 projections with a zoom of 1.3, an angular range of 360° in 5.6° increments, and a duration of 30 seconds per frame. All studies were performed on a large-field-of-view dual-head whole-body camera (DST-XL or DST-Xli, General Electric/Sopha Medical Vision International, Buc, France)

Wilde et al., 2012 [6]	Cross-sectional	20 subjects (14 women; age N/E)	CBCT	Accuitomo (J. Morita MFG Corp., Kyoto, Japan) with the following parameters: 77 kV, 4 mA, scanning time 18 seconds, basis image 184, with a volume of 6 × 6 cm^3^

MDCT: multidetector computed tomography; PAN: panoramic radiography; PER: periapical radiography; CBCT: cone beam computed tomography; SCI: planar scintigraphy; SPECT: single photon emission computed tomography; PET: positron emission tomography; N/S: not specified.

**Table 2:** Objectives, main results, and main conclusions of each selected study.

Authors	Objectives	Main results	Main conclusions
Bianchi et al., 2007 [13]	To verify the radiographic, demographic, and clinical features of BRONJ	MDCT was far superior to PAN in detecting all the radiologic signs. Dental panoramic radiograph may miss the correct diagnosis of sequestration. Intense reaction was often found	PAN was found to be of limited use in assessing BRONJ in patients for whom CT imaging was subsequently ordered

Fleisher et al., 2010 [14]	To verify radiographic changes that develop BRONJ after extraction and the correlation between BRONJ and reduced *serum* CTX values	All patients who had serum CTX levels <150 pg/mL healed successfully after dentoalveolar surgery or after treatment for BRONJ. 83% of patients who had BRONJ exhibited periodontal ligament (PDL) widening associated with extracted teeth, while only 11% who healed normally demonstrated PDL widening	The radiographic PDL widening may be a more sensitive indicator than CTX testing in predicting risk of BRONJ. Minimal surgical intervention may need to be revised to include alternative strategies for the elimination or management of this pathology

Guggenberger et al., 2013 [15]	To compare the extent of changes compatible with BRONJ on MRI, PET/CT, and CBCT of the jaw with clinical preoperative and intraoperative examinations	There were significant differences in BRONJ extent among modalities and examinations (*P* < 0.001).The highest median rank was seen in PET/CT and MRI imaging, followed by intraoperative examinations, CBCT, and preoperative examinations. Preoperative examinations showed significantly less extensive disease than all other modalities/examinations (all *P* < 0.05)	PET/CT and MRI imaging revealed more extensive involvement of BRONJ compared with panoramic views from CBCT and clinical examinations

Rocha et al., 2012 [11]	To compare radiographic alterations in patients taking bisphosphonate with a control group that would permit early diagnosis of BRONJ	Patients treated with zoledronate presented a statistically significant increase in the number of radiographic abnormalities compared with the control group. Female patients presented significantly more alterations than male patients, and the posterior region of the mandible was the most affected region	The use of panoramic radiographs facilitates early identification of bone alterations, which can improve early diagnosis of BRONJ

Stockmann et al., 2010 [16]	To find out the adequate imaging techniques to assess the extent of BRONJ	The detectability of BRONJ was 54% in PAN, 92% for MRI, and 96% for MDCT	MRI and MDCT have a higher detectability than PAN. The relevance of MRI and MDCT for the preoperative assessment of the extent of BRONJ is limited

Takaishi et al., 2010 [17]	To characterize alveolar bone under imminent danger for BRONJ by a radiogrammetric method on the alveolar bone mineral density	The bone mineral density surrounding the osteonecrosis lesions showed distinctly higher density in BRONJ cases compared with age-matched controls. In one subject on bisphosphonate treatment in which two extractions were simultaneously carried out, BRONJ occurred only at the location with extremely high alveolar bone density, but not at the other site with normal density	This method may be useful in detecting a rise of alveolar bone mineral density frequently occurring near the necrotic lesion in subjects with impending risk for BRONJ

Torres et al., 2011 [18]	To compare fractal dimensions (FD) in CBCT exams of patients with BRONJ with a control group and select the best region of interest for detecting bone alterations	The value of the FD in the area of exposed bone was the highest. The odds of being a BRONJ patient versus being a control were six times as high for individuals with a higher FD score at a region of interest in the alveolar process, although the confidence interval was quite wide owing to the small sample size	BRONJ patients had higher FD values than controls at regions close to the alveolar process. FD is a promising tool for detection of bone alterations associated with BRONJ

Torres et al., 2012 [19]	To compare cortical bone measures in CBCT exams of patients with BRONJ with a control group	The cortical bone measurements were significantly higher in cases than in controls. The bone measurements were strongly associated with BRONJ case status	Mandibular cortical bone measurement is a potentially useful tool in the detection of bone dimensional changes caused by bisphosphonates

Treister et al., 2009 [20]	To determine the extent to which clinical and radiographic features of BRONJ are correlated	There was agreement between clinical and radiographic detection. There was equivalency between BRONJ diagnosis and both sclerosis and surface irregularity. The correlation between the number of clinical sites and any radiographic finding was significant in the maxilla (*P* < 0.001) but not in the mandible (*P* = 0.178). The total number of radiographic signs per patient increased with BRONJ stage	Focal panoramic radiographic findings of sclerosis and surface irregularity correlate with clinical sites of BRONJ. This may be a useful and reliable tool to detect early changes of BRONJ or to confirm a clinical diagnosis

Van den Wyngaert et al., 2011 [21]	To identify images that predict the healing of BRONJ	SPECT acquisitions were proved superior over planar images in detecting BRONJ lesions. Quantification of tracer uptake in the BRONJ lesion relative to the unaffected side showed increasing uptake with higher stages of ONJ. The relative ratio of uptake was found to be an independent predictor of BRONJ healing BRONJ stage and relative ratio of uptake were not predictors of the occurrence of BRONJ relapses	Bone scintigraphy in patients with BRONJ is feasible. SPECT acquisitions are preferred over planar images. Relative quantification of tracer uptake provides prognostic information independent of clinical stage that may assist in identifying patients with a poor prognosis

Wilde et al., 2012 [6]	To investigate the prevalence of typical radiological findings of BRONJ in CBCT and the relationship of the imaging findings with the severity of BRONJ sites	The most common imaging findings were cancellous bone destruction and cortical bone erosion and can often be seen in all stages of the disease, including low stages. The prevalence of typical findings such as bone destruction, sequestration, and osteosclerosis seems to decrease with decreasing severity of BRONJ. The occurrence of periosteal new bone formation seems to start in high-stage BRONJ	With the exception of formation of new periosteal bone, all investigated radiological signs can be seen across all stages of BRONJ, and occurrence seems to decrease with decreasing severity of the disease. The radiological signs destruction of the cancellous bone and erosion of the cortical bone were the two most frequent and typical findings for BRONJ in CBCT scans

MDCT: multidetector computed tomography; PAN: panoramic radiography; PER: periapical radiography; CBCT: cone beam computed tomography; SCI: planar scintigraphy; SPECT: single photon emission computed tomography; PET: positron emission tomography.
